# Effect of various g-C_3_N_4_ precursors on the catalytic performance of alkylorganotin-based catalysts in acetylene hydrochlorination

**DOI:** 10.3906/kim-1909-64

**Published:** 2020-04-01

**Authors:** Yibo Wu, Fuxiang LI, Jianwei XUE, Zhiping LV

**Affiliations:** 1 College of Chemistry and Chemical Engineering, Taiyuan University of Technology, Taiyuan China

**Keywords:** alkylorganotin-based catalysts, g-C_3_N_4_, vinyl chloride, acetylene hydrochlorination

## Abstract

A series of alkylorganotin-based catalysts (Sn-g-C_3_N_4_ /AC) was prepared by wet impregnation in ethanol using different g-C_3_N_4_ precursors and alkylorganotin compounds. The structure, texture, surface composition, and adsorption properties of the as-prepared catalysts were extensively characterized. Then, the obtained samples were evaluated for their catalytic performance in hydrochlorination of acetylene. The results provided by the X-ray photoelectron spectroscopy, acetylene temperature-programmed desorption, and HCl adsorption confirmed the nature of the active sites (i.e. Sn-N_x_) involved in the reactant adsorption, and hence in the improved catalytic performance. These active sites were also related to the improved lifetime of alkylorganotin-based catalysts in the hydrochlorination of acetylene. At a constant reaction temperature of 200 °C with an acetylene gas hourly space velocity (C_2_H_2_ -GHSV) of 30 h^-1^ , Sn-g^1^ -C_3_N_4_ /AC-550 exhibited the highest acetylene conversion (~98.0%) and selectivity toward the vinyl chloride monomer (>98.0%). From the catalytic test results, it was reasonably concluded that the hexamethylenetetramine is the most suitable N precursor, as compared to the dicyandiamide and urea, to prepare high-performance catalysts. From the BET specific surface area of fresh and used catalysts, it was suggested that, in contrast to dicyandiamide and urea, hexamethylenetetramine could delay the deposition of coke on alkylorganotin-based catalysts, which is reflected by the extended lifetime.

## 1. Introduction

Owing to the widespread applications of polyvinyl chloride (PVC) in all human activities, there is an increased demand for vinyl chloride monomer (VCM) as precursor for PVC manufacture [1,2]. The hydrochlorination of acetylene is the main technology for producing VCM in several countries, especially in China, due to their rich coal reserves. However, there are several drawbacks of the use of carbon-supported mercuric chloride catalysts for the synthesis of VCM via acetylene hydrochlorination. Specifically, stringent government policies and severe mercury pollution have urged the researchers to explore alternative catalysts [3]. Hence, the design and development of mercury-free catalysts is extensively investigated [4].

In recent years, remarkable progress has been achieved in the development of new catalysts, mainly gold-[5–10], nonprecious-metal- [11-20], and nonmetal-based catalysts [21–24]. Researchers have found that Au-based catalysts with a superior hydrochlorination activity are mainly deactivated due to the reduction of Au3+ to Au0 during acetylene hydrochlorination [5–10]. In addition, the high cost of gold limits the application of Au-based catalysts for the large-scale production of VCM. Among non-precious metal catalysts, the mercury-free catalysts have been developed for VCM production. Carbon-supported tin catalysts with a high catalytic performance [11–14] have attracted little attention compared to copper-based catalysts [15–20]. For instance, Xiong et al. [11] have prepared a tetrametallic supported catalyst (SnCl_4_ -CuCl_2_ -BiCl_3_ -CeCl_3_ /AC) and reported an optimal acetylene conversion of 95.1% (reaction conditions: reaction temperature of 120 °C, space velocity of 90 h^-1^ , *V_HCl_* / *V_C2H2_* = 1.1). Deng et al. [12] have used a trimetallic supported catalyst (SnCl_2_ -BiCl_3_ -CuCl/AC) for the hydrochlorination of acetylene and reported that the deactivation of SnCl_2_ -BiCl_3_ -CuCl/AC is mainly attributed to the loss of tin(IV) chloride. In addition, Guo et al. [13] have prepared a SnCl_2_ -ZnCl_2_ -Tb_4_O_7_ /AC catalyst and reported the highest acetylene conversion of 67.7% (reaction conditions: reaction temperature = 140 °C, *V_HCl_* / *V_C2H2_* = 1.1, C_2_H_2_ -GHSV = 300 h^-1^) . Moreover, researchers have reported that nonmetal elements, including N [21], P [22], S [23], and B [24] can act as active catalysts for acetylene hydrochlorination.

Alkylorganotin compounds have been widely used as stabilizers and catalysts, as well as used in cancer treatment [14, 25–29]. Previous studies have revealed that organotin can catalyze the hydrochlorination of acetylene to obtain vinyl chloride. Moreover, additional performance enhancement of organotin compounds has been achieved by using dicyandiamide additives [14]. Carbon nitride (g-C_3_N_4_) has attracted considerable attention for heterogeneous catalysis, photocatalysis, gas adsorption, and storage due to its high chemical stability and excellent acid and alkali resistance [21,30–38]. Particularly, the Dai’s group has investigated the catalytic performance of g-C_3_N_4_ /AC for acetylene hydrochlorination and reported that the nitrogen enrichment of catalysts can promote the adsorption of hydrogen chloride and thus, significantly increase the activity of this metal-free catalyst [21]. However, the manner in which different g-C_3_N_4_ precursors affect the physicochemical properties of organotin is still not clear. Thus, it is imperative to further examine the use of alkylorganotin-based catalysts for acetylene hydrochlorination.

In this study, hexamethylenetetramine, urea, and dicyandiamide were selected as precursors of g-C_3_N_4_ , and the effect of these precursors on the catalytic properties of alkylorganotin-based catalysts was investigated. The as-obtained catalysts were characterized by X-ray diffraction (XRD), N_2_ adsorption-desorption isotherms, thermogravimetric analysis (TGA), and derivative thermogravimetric (DTG) analysis, X-ray photoelectron spectroscopy (XPS), HCl adsorption, and acetylene temperature-programmed desorption (C_2_H_2_ -TPD). The results indicated that hexamethylenetetramine is the best g-C_3_N_4_ precursor as Sn-N_x_ in alkylorganotin serves as catalytically active sites.

## 2. Results and discussion

### 2.1. Physicochemical properties of g-C_3_N_4_ /AC catalysts

**Figure 1:**
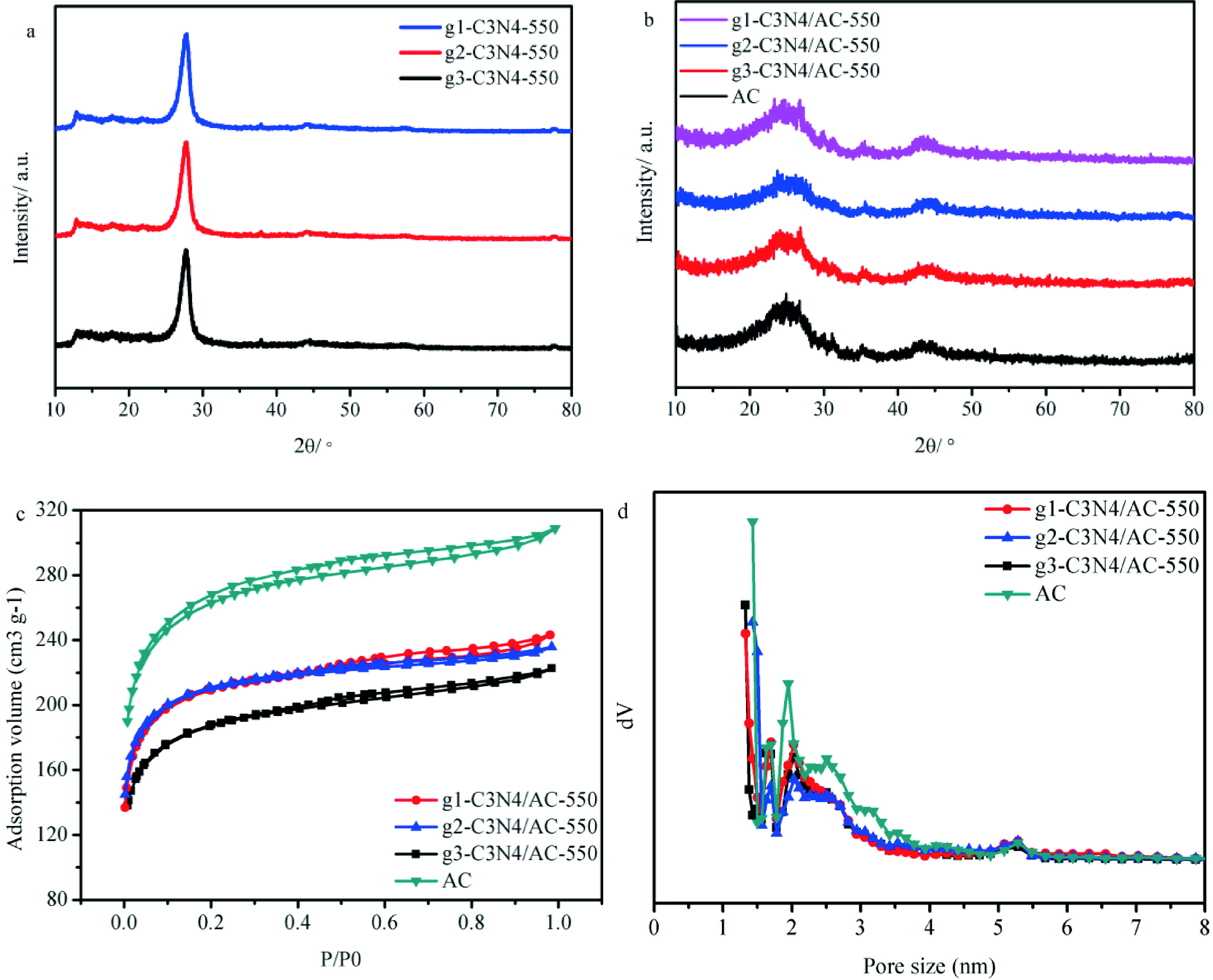
XRD patterns of (a) g-C_3_N_4_ ; (b)g-C_3_N_4_ /AC catalysts, and AC; (c) N_2_ adsorption/desorption isotherms of g-C_3_N_4_ /AC and AC; (d) pore distribution curves of g-C_3_N_4_ /AC catalysts and AC.

According to the previous reports [33,34,37], the hexamethylenetetramine, dicyandiamide and urea were calcinated at 550 °C for 4 h under nitrogen atmosphere to successfully prepare g-C_3_N_4_ . Figure 1a shows the XRD patterns of g^1^ -C_3_N_4_ -550, g_2_ -C_3_N_4_ -550, and g_3_ -C_3_N_4_ -550. Two peaks were observed at 13.2°and 27.5°, respectively [34,38–40]. In the XRD patterns of g^1^ -C_3_N_4_ /AC-550, g_2_ -C_3_N_4_ /AC-550, and g_3_ -C_3_N_4_ /AC-550 (Figure 1b), 3 peaks at around 24.4°, 26.7°, and 43.7°can be observed, which correspond to the (002), (103), and (101) diffraction planes of the graphitic carbon, respectively (PDF#50-0926). Figure 1c displays the N_2_ physisorption isotherms of AC, g^1^ -C_3_N_4_ /AC-550, g_2_ -C_3_N_4_ /AC-550, and g_3_ -C_3_N_4_ /AC-550 samples. According to the IUPAC classification, they belong to the type I isotherms (Figure 1c). Figure 1d illustrates the pore size distribution curves for g-C_3_N_4_ /AC-550 and AC. They reveal the coexistence of micro- and mesopores in all samples. Based on the isotherms and applying specific equations, the values of the textural parameters were calculated, and they are listed in Table 1. Compared with those of bare AC (986 m^2^ g^-1^ , 0.48 cm^3^ g^-1^) , the BET specific surface area and total pore volume of g^1^ -C_3_N_4_ /AC-550 (790 m^2^ g^-1^ , 0.38 cm^3^ g^-1^) , g_2_ - C_3_N_4_ /AC-550 (806 m^2^ g^-1^ , 0.36 cm^3^ g^-1^) , and g_3_ -C_3_N_4_ /AC-550 (701 m^2^ g^-1^ , 0.34 cm^3^ g^-1^) decreased, indicating the successful loading of g-C_3_N_4_ on the AC support (Table 1). The XRD and N_2_ physisorption results revealed that g-C_3_N_4_ is well dispersed on the AC surface.

**Table 1:** Textural properties of g-C_3_N_4_ /AC and AC catalysts.

Sample	S_*BET*_ (m^2^ g^-1^)	S_*micro*_ (m^2^ g^-1^)	S_*meso*_ (m^2^ g^-1^)	V_*total*_ (cm^3^ g^-1^)	V_*micro*_ (cm^3^g^-1^)	Pore size (nm)
AC	986	864	122	0.48	0.36	1.9
g_1_-C_3_N_4_//AC-550	790	699	91	0.38	0.28	1.9
g_2_-C_3_N_4_/AC-550	806	728	78	0.36	0.29	1.8
g_3_-C_3_N_4_//AC-550	701	605	96	0.34	0.25	2.0

### 2.2. Catalytic performance of g-C_3_N_4_ /AC

**Figure 2:**
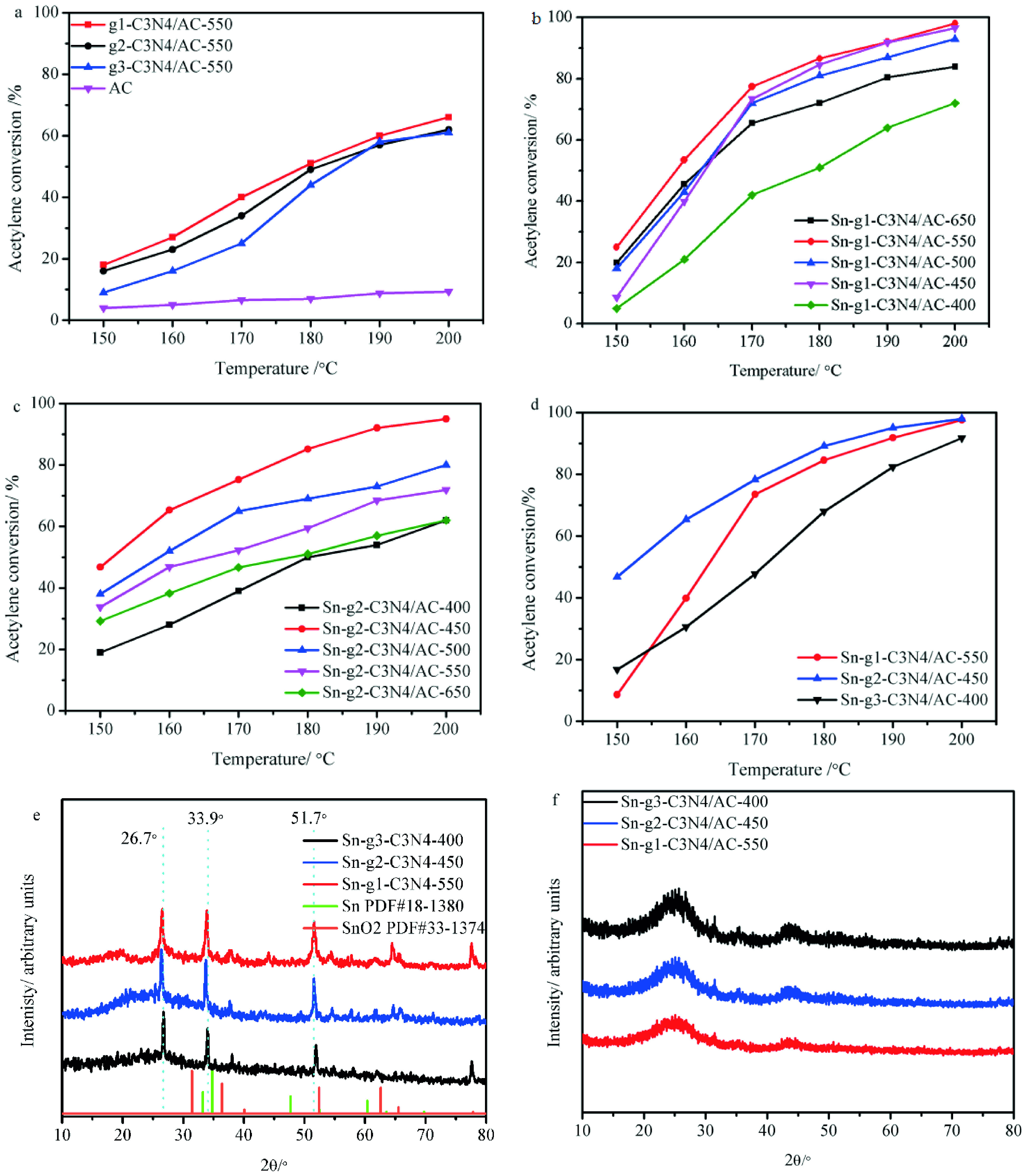
Catalytic performance of (a) g-C_3_N_4_ /AC and AC; (b)Sn-g^1^ -C_3_N_4_/ AC (calcination temperatures of 400, 450, 500, 550, and 600 °C); (c)Sn-g_2_ -C_3_N_4_/ AC (calcination temperatures of 400, 450, 500, 550, and 600 °C); (d) Sn-g^1^ -C_3_N_4_/ AC-550, Sn-g_2_ -C_3_N_4_/ AC-450, and Sn-g_3_ -C_3_N_4_/ AC-400; (e) XRD patterns of Sn-g^1^ -C_3_N_4_ -550, Sng 2 -C_3_N_4_ -450, and Sn-g_3_ -C_3_N_4_ -400, (f) XRD patterns of Sn-g^1^ -C_3_N_4_ /AC-550, Sn-g_2_ -C_3_N_4_ /AC-450, and Sn-g_3_ -C_3_N_4_ /AC-400. Reaction conditions: T = 150 °C–200 °C, C_2_H_2_ -GHSV = 30 h^-1^ , *V_HCl_* / *V_C2H2_* = 1.1

An acetylene conversion of 8.9% was obtained over the AC support at 200 °C (Figure 2a). The g-C_3_N_4_ /AC catalysts exhibited a significantly increased acetylene conversion under the same reaction conditions. That is, conversions of 67.2%, 62.5%, and 61.7% were obtained for g^1^ -C_3_N_4_ /AC-550, g_2_ -C_3_N_4_ /AC-550, and g_3_ -C_3_N_4_ /AC-550, respectively. Notably, high acetylene conversion was obtained for g^1^ -C_3_N_4_ /AC-550, g_2_ -C_3_N_4_ /AC-550, and g_3_ -C_3_N_4_ /AC-550 catalysts, but the values were still less than 90%.

### 2.3. Catalytic performance of Sn-g-C_3_N_4_ /AC

In our previous study [14], the change in the calcination temperature of the g-C_3_N_4_ precursor in the range of 400 °C–650 °C had a positive effect on the hydrochlorination activity of organotin. Thus, to investigate the effect of various g-C_3_N_4_ precursors on the performance of alkylorganotin-based catalysts for acetylene hydrochlorination, the calcination of Sn-g-C_3_N_4_ /AC catalyst precursors was carried out at 400, 450, 500, 550, and 650 °C.

Tables 2 and 3 summarize the BET specific surface areas and total pore volumes of Sn-g-C_3_N_4_ /AC catalysts. Clearly, the BET specific surface areas and total pore volumes of Sn-g-C_3_N_4_ /AC catalysts were lower than those of g-C_3_N_4_ /AC (701–806 m^2^ g^-1^ , 0.38–0.34 cm^3^ g^-1^) , indicating that the pores of g-C_3_N_4_ /AC were filled with alkylorganotin. Hence, BET specific surface areas between 210 and 653 m^2^ g^-1^ are obtained depending on the calcination temperature. First, with the increase in the calcination temperature, the values of the textural properties increased and then decreased, demonstrating the effect of the temperature on the textural properties of Sn-g-C_3_N_4_ /AC. To identify the optimal calcination temperature of Sn-g-C_3_N_4_ /AC, the acetylene hydrochlorination over all samples was performed. Figure 2b depicts the acetylene conversion obtained over Sn-g^1^ -C_3_N_4_ /AC-400, Sn-g^1^ -C_3_N_4_ /AC-450, Sn-g^1^ -C_3_N_4_ /AC-500, Sn-g^1^ -C_3_N_4_ /AC-550, and Sn-g^1^ - C_3_N_4_ /AC-650. With the increase in the reaction temperature from 150 °C to 200 °C, the acetylene conversion over all catalysts clearly increased (Figure 2b and Figure 2c). At an optimal reaction temperature of 200 °C, Sn-g^1^ -C_3_N_4_ /AC-550 exhibited the highest acetylene conversion (97.8%). In addition, the acetylene conversion over g_2_ -C_3_N_4_ /AC obtained at different calcination temperatures decreased in the order of Sn-g_2_ -C_3_N_4_ /AC-450 (97.2%) >Sn-g_2_ -C_3_N_4_ /AC-500 (81.2%) >Sn-g_2_ -C_3_N_4_ /AC-550 (72.1%) >Sn-g_2_ -C_3_N_4_ /AC-650 (63.1%) >Sn-g_2_ -C_3_N_4_ /AC-400 (62.9%) (Figure 2c). These results indicated that the optimal calcination temperatures are 450 °C and 550 °C for Sn-g_2_ -C_3_N_4_ /AC and Sn-g^1^ -C_3_N_4_ /AC, respectively. The hydrochlorination activities of Sn-g^1^ -C_3_N_4_ /AC-550 (97.8%), Sn-g_2_ -C_3_N_4_ /AC-450 (97.2%), and Sn-g_3_ -C_3_N_4_ /AC-400 (92.1%) [14] were higher than that of Sn/AC (89.1%) (Figure 2d) [14]. Therefore, the enhancement in activity of alkylorganotinbased catalysts correlates to the g-C_3_N_4_ precursor, and hexamethylenetetramine is obviously the best g-C_3_N_4_ precursor. Usually, XRD is used to investigate the dispersion of metal species and eliminate the interference of metal agglomeration [18,41]. As shown in Figure 2e, the XRD patterns of Sn-g^1^ -C_3_N_4_ -550, Sn-g_2_ -C_3_N_4_ -450, and Sn-g_3_ -C_3_N_4_ -400 display 3 obvious peaks at 26.7°, 33.9°, and 51.7°, which are characteristic to Sn-g-C_3_N_4_ . However, there are no typical peaks of tin metal (PDF#18-1380) and SnO_2_ phases (PDF#33-1374) in Sn-g-C_3_N_4_ , inferring that the high temperature favoured the formation of Sn-g-C_3_N_4_ . Figure 2f displays the XRD pattern of Sn-g^1^ -C_3_N_4_ /AC-550, Sn-g_2_ -C_3_N_4_ /AC-450, and Sn-g_3_ -C_3_N_4_ /AC-400, which are similar to that of AC. Furthermore, 3 peaks at around 24.4°, 26.7°, and 43.7°can be observed, which correspond to the (002), (103), and (101) diffraction planes of the graphitic carbon, respectively (PDF#50-0926). Particularly, the diffraction peaks of Sn-g-C_3_N_4_ (Figure 2e) were not observed in the diffractograms of Sn-g-C_3_N_4_ /AC samples (Figure 2e, 2f), suggesting that Sn-g^1^ -C_3_N_4_ -550, Sn-g_2_ -C_3_N_4_ -450, and Sn-g_3_ -C_3_N_4_ -400 are well dispersed on the AC surface. In line with the conditions used to perform acetylene hydrochlorination at the industrial level, the reaction temperature and acetylene gas hourly space velocity were controlled in the range of 130 °C–180 °C and 30–50 h^-1^ , respectively [42]. However, when the reaction temperature was 180 °C , the acetylene conversion over the Sn-g-C_3_N_4_ /AC did not achieve ~98%. When the temperature increased to 200 °C, the acetylene conversion over Sn-g-C_3_N_4_ /AC increased to ~98%, which is close to the activity of HgCl_2_/AC. Therefore, the stability of catalysts was tested in acetylene hydrochlorination at 200 °C.

**Table 2:** Textural properties of Sn-g^1^ -C_3_N_4_ /AC at different calcination temperatures.

Sample	S_*BET*_ (m^2^g^-1^)	S_*micro*_ (m^2^g^-1^)	S_*meso*_ (m^2^g^-1^)	V_*total*_ (cm^3^g^-1^)	V_*micro*_ (cm^3^g^-1^)	Pore size (nm)
Sn-g1-C_3_N_4_/AC-400	210	151	59	0.13	0.07	2.1
Sn-g1-C_3_N_4_/AC-450	577	524	53	0.27	0.10	1.9
Sn-g1-C_3_N_4_/AC-500	609	550	59	0.28	0.11	1.9
Sn-g1-C_3_N_4_/AC-550	650	559	91	0.31	0.23	1.9
Sn-g1-C_3_N_4_/AC-650	605	550	55	0.27	0.10	2.0

**Table 3:** Textural properties of Sn-g_2_ -C_3_N_4_ /AC at different calcination temperatures.

Sample	S_*BET*_ (m^2^g^-1^)	S_*micro*_ (m^2^g^-1^)	S_*meso*_ (m^2^g^-1^)	V_*total*_ (m^2^g^-1^)	V_*micro*_ (m^2^g^-1^)	Pore size (nm)
Sn-g_2_-C_3_N_4_/AC-400	388	320	68	0.21	0.13	2.0
Sn-g_2_-C_3_N_4_/AC-450	526	434	92	0.27	0.18	1.9
Sn-g_2_-C_3_N_4_/AC-500	585	493	92	0.29	0.21	1.9
Sn-g_2_-C_3_N_4_/AC-550	541	482	59	0.26	0.20	1.9
Sn-g_2_-C_3_N_4_/AC-650	653	594	59	0.30	0.25	1.8

### 2.4. Stability of Sn-g-C_3_N_4_ /AC and Sn/AC catalysts

**Figure 3:**
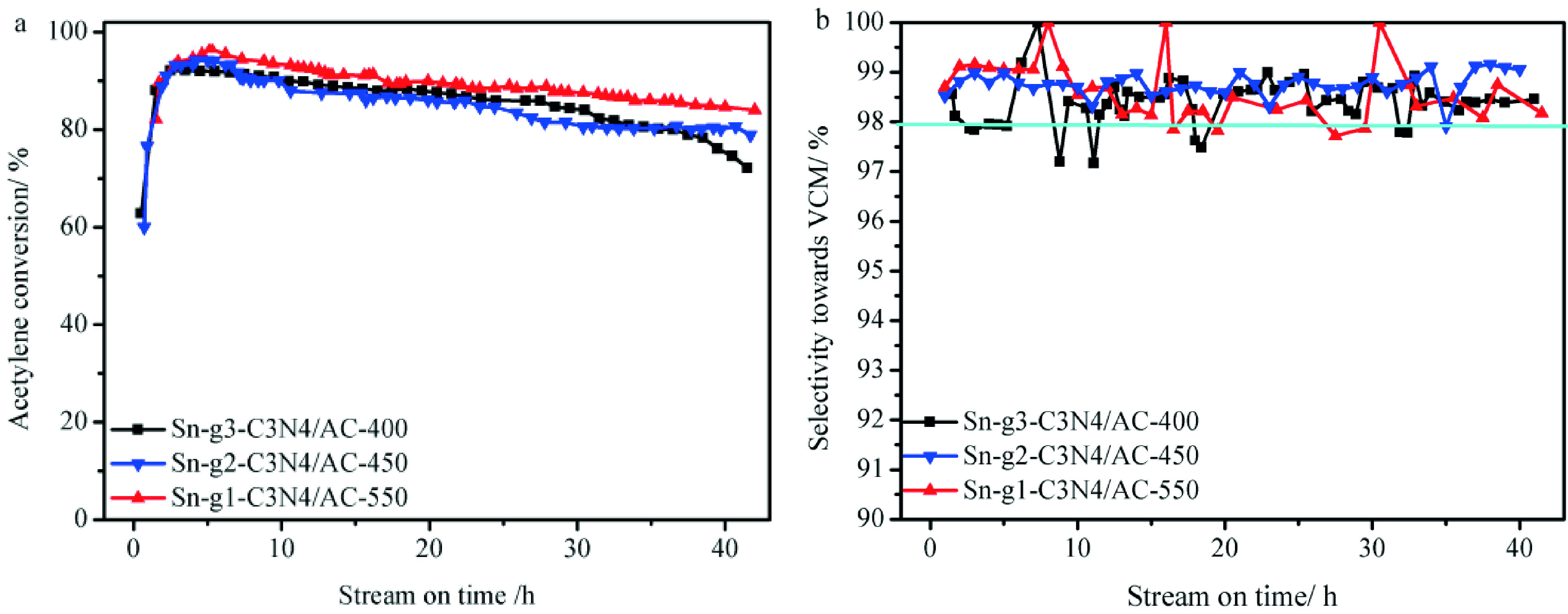
(a) Activity and (b) selectivity toward VCM of alkylorganotin-based catalysts depending on the g- C_3_N_4_ precursor and calcination temperature. Reaction conditions: T = 200 °C, C_2_H_2_ GHSV = 30 h^-1^ , *V_HCl_* / *V_C2H2_* = 1.1

Next, stability experiments were performed for Sn-g^1^ -C_3_N_4_ /AC-550, Sn-g_2_ -C_3_N_4_ /AC-450, and Sn-g_3_ -C_3_N_4_ /AC-400. Figure 3a shows the obtained results. After 14 h of reaction, the acetylene conversion of Sn-g^1^ -C_3_N_4_ /AC-550 was 97.8%, which decreased to 82.1% after 40 h. The acetylene conversion of Sn-g_2_ -C_3_N_4_ /AC-450 and Sn-g_3_ -C_3_N_4_ /AC-400 gradually decreased from 97.2% and 92.1% to 77.5 and 72.1%, respectively, after 40 h of reaction. By comparison, Sn/AC only exhibited an acetylene conversion of 49.5% after 40 h of reaction [14]. Sn-g^1^ -C_3_N_4_ /AC-550, Sn-g_2_ -C_3_N_4_ /AC-450, and Sn-g_3_ -C_3_N_4_ /AC-400 were highly selective toward VCM (>98.0%, Figure 3b). Moreover, the selectivity did not change over the entire reaction period. After the reaction, the BET specific surface area and pore volume of the used catalysts decreased in comparison with those of the fresh catalysts (Table 4) due to the deposition of coke on the catalyst surface. Thus, the percentage decrease in the BET specific surface area of Sn-g^1^ -C_3_N_4_ /AC-550, Sn-g_2_ -C_3_N_4_ /AC-450, Sn-g_3_ -C_3_N_4_ /AC-400, and Sn/AC are 54, 68, 77, and 79%, respectively. This phenomenon is mainly attributed to the polymerization of acetylene and vinyl chloride during acetylene hydrochlorination [43–48]. Consequently, the g-C_3_N_4_ precursor may prevent the loss of the BET specific surface area of alkylorganotin-based catalysts in the hydrochlorination of acetylene, thereby extending the lifetime of the alkylorganotin-based catalysts. Furthermore, from this point of view, among the 3 g-C_3_N_4_ precursors, the hexamethylenetetramine is proved to be the optimum one.

**Table 4:** Textural properties of fresh and used Sn-g-C_3_N_4_ /AC catalysts (after 40 h).

Sample	S_*BET*_ (m^1^ g^-1^)		S_*BET*_ loss (%)	V (cm^3^ g^-1^)		Pore size (nm)	
	fresh used			fresh	used	fresh	used
AC	987	-	-	0.36	-	1.9	1.9	-
Sn/AC-200	243	49	79	0.15	0.02	1.9	1.9	4.7
Sn-g_1_-C_3_N_4_/AC-550	650	295	54	0.31	0.17	1.9	1.9	2.2
Sn-g_2_-C_3_N_4_/AC-450	526	168	68	0.27	0.11	2.0	2.0	4.0
Sn-g_3_-C_3_N_4_/AC-400	233	53	77	0.19	0.05	2.3	2.3	4.4

### 2.5. Characterization of the active sites of Sn-g-C_3_N_4_ /AC catalysts

#### 2.5.1. Chemical states of Sn and N

**Figure 4:**
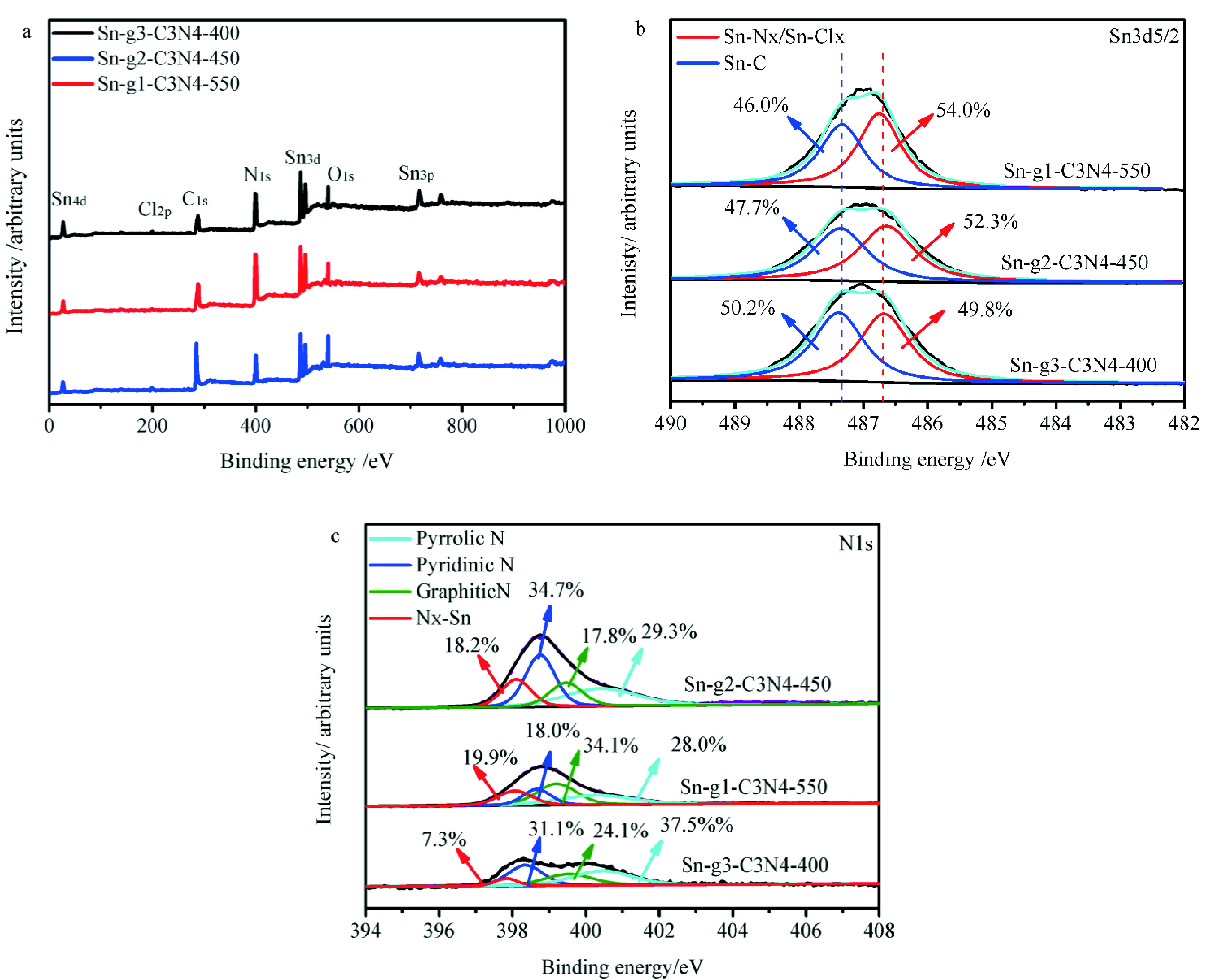
(a) Survey XPS spectra of the Sn-g-C_3_N_4_ catalysts. High-resolution (b) Sn3d and (c) N1 spectra of different Sn-g-C_3_N_4_ catalysts.

XPS was employed to investigate the chemical states of Sn, N, C, and Cl on the Sn-g^1^ -C_3_N_4_ -550, Sn-g_2_ -C_3_N_4_ -450, and Sn-g_3_ -C_3_N_4_ -400 catalyst surfaces. Sn3d , C1s , N1s , and Cl1s signals were detected in all 3 samples (Figure 4a and Table 5).

The Sn3d5/2 signal was fitted with 2 peaks (Figure 4b). The peak at 487.5 eV corresponds to Sn-C [49,50] while the one at 486.8 eV corresponds to Sn-N_x_ (486.7eV) [51] and/or Sn-Cl_x_ (486.9 eV) in Sn-g-C_3_N_4_ /AC, which indicates bond formation between Sn^4+^ and N/Cl [52–54]. According to the relative peak areas (Table 6), the relative contents of Sn-N_x_ /Sn-Cl_x_ in the fresh Sn-g^1^ -C_3_N_4_ -550, Sn-g_2_ -C_3_N_4_ -450, and Sn-g_3_ -C_3_N_4_ -400 catalysts were 2.25%, 2.00%, and 1.64%, respectively. Table 6 summarizes the quantitative results for the N species, and they suggest that the alkylorganotins, which promote the formation of Sn-N_x_ bonds, hinder the loss of Sn during the preparation of catalysts. Compared with the Sn-g_2_ -C_3_N_4_ -450 and Sn-g_3_ -C_3_N_4_ -400, Sn-g^1^ -C_3_N_4_ -550 has a higher content of Sn-N_x_ /Sn-Cl_x_ . The four individual peaks observed at ~397.7, 398.4, 399.4, and 401.0 eV (Figure 4c) correspond to the coexistence of N_x_-Sn, pyridinicN, graphitic N, and pyrrolicN, respectively in all 3 catalysts (Figure 4c and Table 7) [24, 52–57]. The N_x_-Sn content decreased in the order of Sn-g^1^ -C_3_N_4_ -550 (9.17 wt%) >Sn-g_2_ -C_3_N_4_ -450 (6.65 wt%) >Sn-g_3_ -C_3_N_4_ -400 (3.06 wt%), which is in well agreement with the catalytic activity trend (Table 7). The XPS results confirmed that Sn-N_x_ sites play a significant role in the improvement of the hydrochlorination performance of the catalysts. Moreover, compared with the catalysts prepared with urea and dicyandiamide, the catalyst prepared with hexamethylenetetramine (Sn-g^1^ -C_3_N_4_ -550) displays the highest N_x_-Sn content.

**Table 5:** Surface elemental compositions of different Sn-g-C_3_N_4_ as determined by XPS.

Sample	Sn (wt%)	N (wt%)	O (wt%)	Cl (wt%)	C (wt%)
Sn-g_1_-C_3_N_4_-550	4.17	46.07	6.04	1.40	42.32
Sn-g_2_-C_3_N_4_-450	3.83	36.56	9.48	1.67	48.46
Sn-g_3_-C_3_N_4_-400	3.30	41.96	6.23	1.06	47.45

**Table 6:** Contents of Sn^4+^ and Sn-N_x_ /Sn-Cl_x_ in different Sn-g-C_3_N_4_ catalysts.

Sample	Total Sn (wt%)	Sn/C (wt%)	Sn-N_x_/Sn-Cl_x_ (wt%)
Sn-g_1_-C_3_N_4_-550	4.17	1.92	2.25
Sn-g_2_-C_3_N_4_-450	3.83	1.83	2.00
Sn-g_3_-C_3_N_4_-400	3.30	1.66	1.64

**Table 7:** Contents of pyridinic N, graphiniticN, pyrrolicN, and Sn-N_x_ in different Sn-g-C_3_N_4_ catalysts.

Sample	Total N (wt%)	Pyridinic N (wt%)	Graphitic N (wt%)	Pyrrolic N (wt%)	Sn-N_x_ (wt%)
Sn-g_1_-C_3_N_4_-550	46.07	15.99	7.41	13.50	9.17
Sn-g_2_-C_3_N_4_-450	36.56	6.58	13.09	10.24	6.65
Sn-g_3_-C_3_N_4_-400	41.96	13.05	10.11	15.74	3.06

#### 2.5.2. Thermal stability of Sn-g-C_3_N_4_ /AC

**Figure 5:**
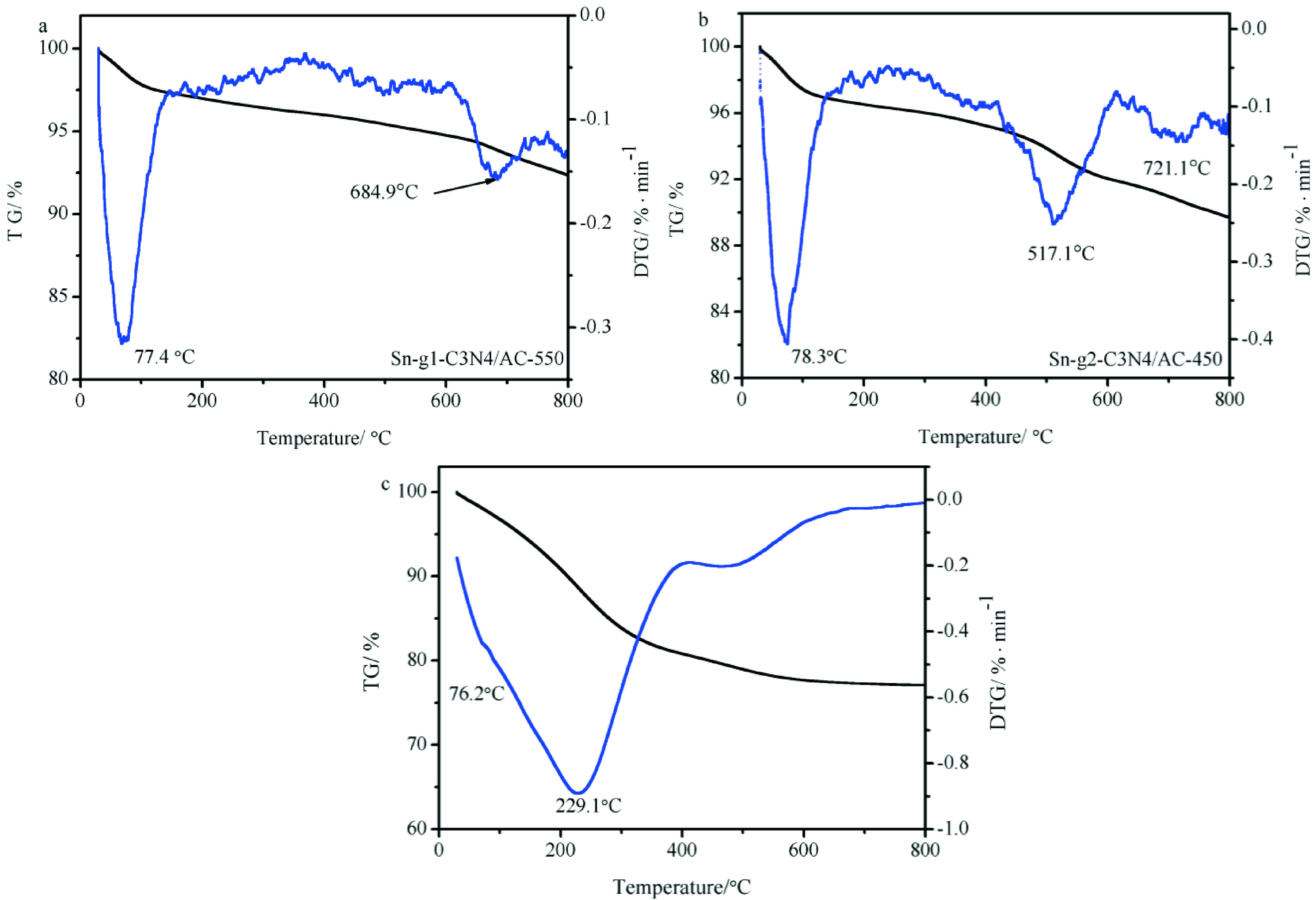
TG and DTG curves of (a) Sn-g^1^ -C_3_N_4_ /AC-550; (b) Sn-g_2_ -C_3_N_4_ /AC-450, and (c) Sn-g_2_ -C_3_N_4_ /AC-400.

To investigate the thermal stability of Sn-g^1^ -C_3_N_4_ /AC-550, Sn-g_2_ -C_3_N_4_ /AC-450, and Sn-g_3_ -C_3_N_4_ /AC- 400, TGA curves were recorded under nitrogen. Figures 5a, 5b, and 5c show the TG-DTG curves recorded between 25 °C and 800 °C for the investigated catalysts. All 3 catalysts exhibited a similar weight loss trend. DTG peaks observed at ~684.9 °C (Sn-g^1^ -C_3_N_4_ /AC-550), 517.1 °C (Sn-g_2_ -C_3_N_4_ /AC-450), and 299.0 °C (Sn-g_3_ -C_3_N_4_ /AC-400) suggested that the thermal stability of Sn-g^1^ -C_3_N_4_ /AC-550 is higher than those of Sn-g_2_ -C_3_N_4_ /AC-450 and Sn-g_3_ -C_3_N_4_ /AC-400.

### 2.6. C_2_H_2_ -TPD and HCl adsorption

**Figure 6:**
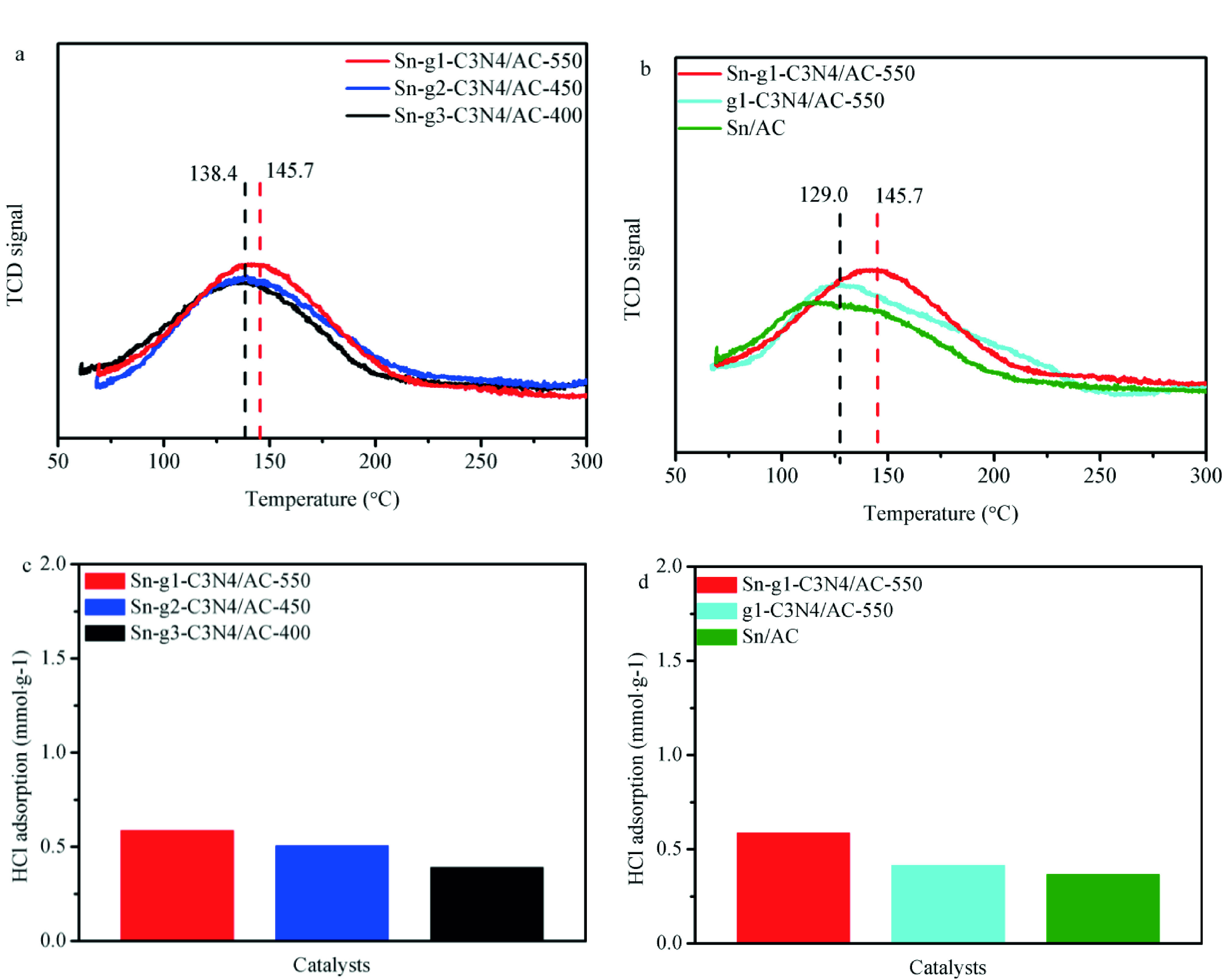
C_2_H_2_ -TPD profiles of different alkylorganotin-based catalysts. (a) g^1^ -C_3_N_4_ /AC-550, g_2_ -C_3_N_4_ /AC-450, and g_3_ -C_3_N_4_ /AC-400; (b) Sn-g^1^ -C_3_N_4_ /AC-550, g^1^ -C_3_N_4_ /AC-550, and Sn/AC; (c) hydrogen chloride adsorption ong^1^ -C_3_N_4_ /AC-550, g_2_ -C_3_N_4_ /AC-450, and g_3_ -C_3_N_4_ /AC-400; (d) Sn-g^1^ -C_3_N_4_ /AC-550, g^1^ -C_3_N_4_ /AC-550, and Sn/AC.

Both Sn^4+^ and HCl as electron-acceptors do not react with each other [58], inferring that alkylorganotin firstly prefers to interact with C_2_H_2_ in acetylene hydrochlorination and then reacts with HCl to generate vinyl chloride. Therefore, reactant adsorption of catalysts behaves a significant impact on catalytic performance. As shown in Figure 6a, the acetylene adsorption capacity follows the order of Sn-g^1^ -C_3_N_4_ -550 >Sn-g_2_ -C_3_N_4_ -450 >Sn-g_3_ - C_3_N_4_ -400, suggesting that compared to Sn-g_2_-C_3_N_4_/AC-450 and Sn-g_3_-C_3_N_4_/AC-400, Sn-g^1^-C_3_N_4_/AC-550 exhibits the highest acetylene adsorption capacity. The temperature of acetylene adsorption on Sn-g^1^-C_3_N_4_/AC-550 (145.7 °C) is higher than those at which the acetylene was adsorbed on Sn-g_2_-C_3_N_4_/AC-450 (138.5 °C) and Sn-g_3_-C_3_N_4_/AC-400 (138.4 °C) (Figure 6a). Therefore, the strength of acetylene adsorption on Sn-g^1^-C_3_N_4_/AC-550 is higher as compared to those on Sn-g_2_-C_3_N_4_/AC-450 and Sn-g_3_-C_3_N_4_/AC-400. The C_2_H_2_ - TPD profiles of Sn-g^1^ -C_3_N_4_ /AC-550, g^1^ -C_3_N_4_ /AC-550, and Sn/AC are illustrated in Figure 6b. Although Sn-C and Sn-Cl_x_ co-exist in Sn/AC and Sn-g^1^ -C_3_N_4_ /AC-550, the last sample exhibits higher acetylene adsorption capacity than Sn/AC-550. Furthermore, Figure 6b shows that the acetylene adsorption capacity and the adsorption strength both increased in Sn-g^1^ -C_3_N_4_ /AC-500 samples, improvement was associated with the existence of Sn-N_x_ in these catalysts. As shown in Table 6, hexamethylenechloride as nitrogen precursor can stabilize the content of Sn and thus, an enhanced catalytic performance was obtained for this sample in comparison with those of the catalysts obtained with urea and dicyandiamide. The corresponding catalytic results are depicted in Figure 2d.

A previous study [59] has reported that hydrogen chloride adsorption is the rate determining step of the reaction. Thus, the adsorption of hydrogen chloride on the catalyst surface is the key factor in acetylene hydrochlorination. The amounts of hydrogen chloride adsorbed on Sn-g^1^ -C_3_N_4_ /AC-550, Sn-g_2_ -C_3_N_4_ /AC-450, and Sn-g_3_ -C_3_N_4_ /AC-400 were 0.59, 0.51, and 0.40 mmol g^-1^ , respectively (Figure 6c). Moreover, the amount of hydrogen chloride adsorbed on the catalysts with various compositions decreased in the order of Sng 1 -C_3_N_4_ /AC-550 (0.59 mmol g^-1^) >g^1^ -C_3_N_4_ /AC-550 (0.41 mmol g^-1^) >Sn/AC (0.37 mmol g^-1^) (Figure 6d). The higher hydrogen chloride adsorption capacity of Sn-g^1^ -C_3_N_4_ /AC-550 is attributed to the coexistence of pyridinic N [24] and Sn-N_x_ sites.

## 3. Conclusions

According to the previous study, MF-600 with 94.5% acetylene conversion was prepared using melamine and toxic formaldehyde [57]. In addition, Sn/AC and g-C_3_N_4_ /AC exhibited acetylene conversion of 89.1% and 76.5%, respectively, in acetylene hydrochlorination [14,21]. To meet the industrial requirement of activity and the green route development of chemical industry and further study the properties of Sn-based catalysts during acetylene hydrochlorination, in this study, Sn-g-C_3_N_4_ /AC as novel nonprecious metal-based catalyst was prepared with alkylorganotin and g-C_3_N_4_ precursors by wet impregnation, as well as exhibited higher acetylene conversion (97.8%). The excellent performance was mainly attributed to the coexistence of Sn-N_x_ and pyridinic N in Sn-g-C_3_N_4_ /AC catalysts. The results of XPS, TG-DTG, C_2_H_2_ -TPD, HCl adsorption, N_2_ physisorption, and stability tests confirmed that the g-C_3_N_4_ precursors can stabilize the Sn species provided by the alkylorganotin precursors, improve the thermal stability of Sn species and adsorption capacity of the resulted catalysts. In addition, the coke deposition is delayed over these alkylorganotin-based catalysts, which is favourable for a longer lifetime, as compared with Sn/AC. Among the 3 g-C_3_N_4_ precursors, hexamethylenetetramine, having higher nitrogen content, proved to be the best g-C_3_N_4_ precursor.

## 4. Materials and methods

### 4.1. Chemicals

Tin(IV) chloride (99.0%) was purchased from Aladdin Co., Ltd. Dioctyldichlorotin (98.0%) was purchased from Energy Chemical Co., Ltd. Hexamethylenetetramine (N1 , 99.0%), urea (N_2_ , 99.0%), and dicyandiamide (N3 , 99.0%) were purchased from the Tianjin Guangfu Fine Chemical Industry Research Institute. Carbon supports were purchased from Shanxi Xinhua Chemical Company. All reagents were used as received without any further purification.

### 4.2. g-C_3_N_4_ /AC preparation

Carbon supports were initially washed with 0.01 mol L−1 HCl to remove the impurities and then dried overnight at 100 °C in an oven. The obtained carbon material was denoted as AC.

N1 (2.0 g) and AC (14.0 g) were mixed with ethanol (100 mL) and then stirred at 80 °C for 3.5 h. Afterwards, the mixture was dried overnight at 100 °C. Finally, the sample was subjected to calcination at 550 °C for 4 h to obtain the g^1^ -C_3_N_4_ /AC-550 sample. The other 2 catalysts (g_2_ -C_3_N_4_ /AC-550 and g_3_ - C_3_N_4_ /AC-550, respectively) were prepared in a similar way.

### 4.3. Sn-g-C 3 N4 /AC preparation

Sn-g-C_3_N_4_ /AC was prepared according to one of our previous studies by using an optimum molar ratio of 1.6:1.0 (SnCl_4_ to C_16_H_34_Cl_2_Sn) [14]. First, organotin compounds (SnCl_4_ and C_16_H_34_Cl_2_Sn, 4.0 g) and 2.0 g N1 were dissolved in 100 mL of ethanol and stirred at 80 °C for 30 min. Secondly, AC (14.0 g) was added to the solution and magnetically stirred for 3 h. Next, the obtained sample was dried overnight at 100 °C in an oven. Finally, solids were subjected to calcination at 450 °C for 4 h under nitrogen to obtain the final catalyst, labelled as Sn-g^1^ -C_3_N_4_ /AC-450. The other 2 catalysts (Sn-g_2_ -C_3_N_4_ /AC and Sn-g_3_ -C_3_N_4_ /AC, respectively) were prepared in a similar way.

### 4.4. Catalyst characterization

Powder X-ray diffraction patterns were recorded on a Shimadzu XRD-6000 instrument with Cu-Kα radiation operated at 40kV.

The textural properties of samples were analysed by nitrogen physisorption on a Nova2000e instrument (Quantachrome) after degassing the samples at 150 °C for 3h.

Thermogravimetric analysis was performed on a NETZSCH STA 449F3 analyser. The temperature was increased from room temperature to 800 °C at a heating rate of 15°C min^-1^ under air at a flow rate of 30 mL min^-1^ .

X-ray photoelectron spectroscopy was performed with an EscaLab 250Xi spectrometer using a monochromatic Al Kα source.

Acetylene temperature-programmed desorption (C_2_H_2_ -TPD) was performed with a FINESORB-3010 chemisorption analyser. TPD experiments were carried out with ~50 mg of sample, which was first treated at 200 °C for 1.5 h under Ar. After cooling, it was continually flushed with C_2_H_2_ at a flow rate of 25 mL min^-1^ for 1 h and then heated from room temperature to 500 °C at a heating rate of 10 °C min^-1^ .

Hydrogen chloride adsorption experiments were performed in a fixed-bed reactor. Catalysts were initially pretreated at 200 °C for 1 h under Ar. Then, hydrogen chloride was fed into the reactor at a flow rate of 30 mL min^-1^ for 1 h. Finally, the samples were heated from 200 °C to 650 °C under Ar, and desorbed hydrogen chloride was removed using deionized water (1000 mL). The amount of hydrogen chloride in the final solution was evaluated by titration [60].

### 4.5. Catalyst performance

The performance of catalysts (4.0 mL) was tested in a fix-bed reactor (d = 10mm). To activate the catalyst and remove the air and physisor bed water, hydrogen chloride gas was initially passed through the reaction system for 40 min. Then, a mixture of hydrogen chloride and acetylene ( *V_HCl_* / *V_C2H2_* = 1.1) was passed through the reactor (reaction temperature = 150 °C–200 °C, C_2_H_2_ -GHSV = 30 h^-1^) . The unreacted hydrogen chloride in the product gas was adsorbed on limestone. The clean gas was then analysed online using a gas chromatograph equipped with a GDX-301column and TCD.
